# The 100 top-cited articles in menopausal syndrome: a bibliometric analysis

**DOI:** 10.1186/s12978-024-01770-9

**Published:** 2024-04-08

**Authors:** Zishan Jin, Chuanxi Tian, Mengjiao Kang, Shiwan Hu, Linhua Zhao, Wei Zhang

**Affiliations:** 1 Beijing University of Chinese Medicine, Beijing, 100029 China; 2 Institute of Metabolic Diseases, Guang’anmen Hospital, China Academy of Chinese Medical Sciences, Beijing, 100053 China; 3 Gansu University of Chinese Medicine, Lanzhou, 730000 Gansu China

**Keywords:** Menopausal syndrome, Systematic review, Bibliometric analysis

## Abstract

**Background:**

Significant scientific research has been conducted concerning menopausal syndrome(MPS), yet few bibliometric analyses have been performed. Our aim was to recognise the 100 most highly cited published articles on MPS and to analytically evaluate their key features.

**Methods:**

To identify the 100 most frequently cited articles, a search was conducted on Web of Science using the term 'menopausal syndrome'. Articles that matched the predetermined criteria were scrutinised to obtain the following data: citation ranking, year of publication, publishing journal, journal impact factor, country of origin, academic institution, authors, study type, and keywords.

**Results:**

The publication period is from January 1, 2000, to August 31, 2022. The maximum number of citations was 406 and in 2012. The median citations per year was 39.70. Most of the articles focused on treatment and complications. These articles were published in 36 different journals, with the Journal of MENOPAUSE having published the greatest number (14%). Forty-eight articles (48%) were from the United States, with the University of Pittsburgh being the leading institute (9%). Joann E. Manson was the most frequent first author (n = 6). Observational studies were the most frequently conducted research type (n = 53), followed by experimental studies (n = 33). Keyword analysis identified classic research topics, including genitourinary syndrome of menopause, bone mineral density (BMD), and anti-mullerian hormone (AMH) loci.

**Conclusion:**

Using bibliometrics, we conducted an analysis to identify the inadequacies, traditional focal points, and potential prospects in the study of MPS across current scientific areas. Treatment and complications are at the core of MPS research, whereas prediction and biomarkers have less literature of high quality. There is a necessity for innovative analytical metrics to measure the real effect of these papers with a high level of citation on clinical application.

## Introduction

Menopausal syndrome (MPS) is a series of disorders of autonomic nervous system, accompanied by neuropsychological symptoms, caused by estrogen fluctuation or decrease in the period of before and after menopause. The core reason lies in the gradual decline of ovarian function, including natural menopause and artificial menopause. In particular, this is not the end of old age, but the beginning of senescence in biological age. The life expectancy of women further increased to 80.88 years [1], which means the irreversible state of low estrogen will last for at least 30 ~ 40 years. 1/3 or even longer time will be in the post-menopausal senescence state for women, and the more incidence of complication will be significantly increased [2, 3]. We conduct a comprehensive statistical overview of all literature in the MPS field, providing a broad perspective from a macroscopic viewpoint to understand the research trends and knowledge structure of this area, presenting an overview of the research landscape across the entire field. Aims to revealing the milestones and discovering the research direction, it evaluates the status quo of MPS by quantitatively analyzing the selected articles in this study.

## Methods

### Inclusion criteria and exclusion criteria

Electronic database of Web of Science was used to query the MPS related articles. The retrieval strategy was TS = (‘perimenopausal syndrome’) OR TS = (‘postmenopausal syndrome’) OR TS = (‘premenopausal Syndrome’) OR TS = (‘menopausal syndrome’) OR TS = (‘Climacteric syndrome’). The inclusion criteria included the core subject as MPS in ‘journal article’, or ‘original article’. The publication year restricted from January 1st, 2000 to August 31st, 2022. The exclusion criteria included MPS articles in position papers, guidelines, reviews, meta-analysis, letters, or editorials. All the included articles independently examined and verified by two reviewers (W. Zhang and Z. Jin). Discrepancies were resolved by consensus with the third senior reviewer (L. Zhao).

### Citation analysis

This study was strictly operated following the previous bibliometric literatures [4–6]. The data was extracted, besides the keywords, which also including type of the study, authors, country and institution, title, quote frequency, publication year, journal. All the words must be uniformly standardized. And, little information provided words, such as women, disease, etc., has been eliminated for the subsequent analysis. The most important information was focus on the co-relationship of authorship, institution, country and keywords in this database. Additionally, a systematic literature review was carried out around keywords.

### Statistical analysis and bibliometric network construction

All the statistical analysis was performed by the IBM SPSS Statistics (version 26.0), following the continuous variables with normal distribution expressed as the means with standard deviation (SD). VOSviewer software (version 1.6.18, Leiden University Center for Science and Technology Studies, Leiden, Netherlands) and CiteSpace software (version 6.1.3,) was used to structure the bibliometric network.

## Results

The 100 top-cited articles were summarized in descending order according to the number of total citations (Table 1). With the publishing date from 2012 to 2022, the largest number of literature published in the year 2012, 2013, 2014, respectively. Among 100 top-cited articles, 53 publications were observational study. Next up were experimental study(n = 33), in vivo study(n = 10), in vitro study(n = 3). Of 36 different journals, MENOPAUSE (n = 14), J CLIN ENDOCR METAB (n = 12), MATURITAS(n = 6) and JAMA related journal (n = 6) published the greatest number of these articles. The median times cited all databases of MPS was 111 (IQR, 98.25–147.5). The maximum of total citations was 406 in BRIT MED J, which reported by Hvidovre Univ Hosp of Denmark in 2012. And, the median of average times per year of cited all databases of MPS was 14.07 (IQR, 10.44–20.07). And, the maximum of average times per year was 62.5 in CELL, which reported by the scientific researchers of Chinese Academy of Sciences of China in 2020.

**Table 1:** The top 100 cited articles in menopausal syndrome

Concern factors	Stage of menopause	Country	Citation	Average citation	Publication Year	Fist institute	Joural	Study type	References
Cardiovascular disease (CVD)	Postmenopausal women (aged 45–58 years)	Denmark	406	39.70	2012	University of Copenhagen	BRIT MED J	Experimental study	[19]
Cardiovascular disease (CVD)	Postmenopausal women	USA	369	59.33	2016	University of Southern California	NEW ENGL J MED	Experimental study	[99]
Breast cancer	Premenopausal women	USA	330	46.29	2015	Cleveland Clinic Foundation	NEW ENGL J MED	Experimental study	[100]
Vulvovaginal atrophy (VVA)	Postmenopausal women	USA	270	29.33	2013	Case Western Reserve University	J SEX MED	Observational study	[13]
Vulvovaginal atrophy (VVA)	Postmenopausal women (aged 55–65 years)	ITALY	227	22.50	2012	IRCCS Fondazione San Matteo;University of Pavia	CLIMACTERIC	Observational study	[101]
Cardiovascular disease (CVD)	Menopausal women (aged 42–58 years)	USA	216	26.75	2014	University of California System	ANN INTERN MED	Experimental study	[102]
Age at natural menopause (ANM)	Age at natural menopause (ANM)	UK	215	29.29	2015	University of Cambridge	NAT GENET	Observational study	[103]
Glycolipid metabolism		USA	202	21.89	2013	US Department of Veterans Affairs	DIABETES	In vivo study (animal)	[104]
Vulvovaginal atrophy (VVA)	Postmenopausal women (aged 59.6 ± 5.8 years)	ITALY	200	24.00	2014	Vita-Salute San Raffaele University	CLIMACTERIC	Experimental study	[15]
Age at natural menopause (ANM)	Initially premenopausal and early perimenopausal women	USA	198	21.11	2013	University of California System	AM J EPIDEMIOL	Observational study	[105]
Cognitive function	Surgical menopause	USA	194	23.63	2014	Brigham and Women's Hospital	NEUROLOGY	Observational study	[106]
Age at natural menopause (ANM)	Late reproductive age women	USA	183	17.90	2012	University of Pennsylvania	J CLIN ENDOCR METAB	Observational study	[107]
Obesity and osteoporosis		USA	173	31.60	2017	Icahn School of Medicine at Mount Sinai	NATURE	In vivo study (animal)	[108]
Alzheimer's disease	Menopausal women	USA	172	16.70	2012	Johns Hopkins University	NEUROLOGY	Observational study	[109]
Cardiovascular disease (CVD) and stroke	Early menopause (aged 45–84 years)	USA	170	16.30	2012	University of Alabama System	MENOPAUSE	Observational study	[110]
Breast cancer	Premenopausal women	UK	161	37.75	2018	University of London	JAMA ONCOL	Observational study	[111]
HPV	Premenopausal women	South Korea	158	16.56	2013	Seoul National University	PLOS ONE	Observational study	[112]
The protection of primordial follicle oocyte		Australia	157	15.30	2012	Walter & Eliza Hall Institute	MOL CELL	In vivo study (animal)	[113]
Vulvovaginal atrophy (VVA)	Postmenopausal women	ITALY	154	21.14	2015	Vita-Salute San Raffaele University	CLIMACTERIC	Experimental study	[14]
Age at natural menopause (ANM)		Netherlands	152	16.78	2013	Utrecht University	J CLIN ENDOCR METAB	Observational study	[114]
AMH level	Premenopausal women	Netherlands	152	16.78	2013	Utrecht University	J CLIN ENDOCR METAB	Observational study	[115]
Cardiovascular disease	Premenopausal, early perimenopausal, late perimenopausal, early or late postmenopausal	USA	151	13.80	2012	University of Colorado System	J CLIN ENDOCR METAB	Observational study	[116]
Ovarian aging		CHINA	151	62.50	2020	Chinese Academy of Sciences	CELL	In vitro study(animal)	[117]
Stroke is associated with blood pressure	Postmenopausal women	USA	148	14.80	2012	Columbia University	HYPERTENSION	Observational study	[118]
Vaginal atrophy	Postmenopausal women	ITALY	148	20.57	2015	University of Insubria	LASER MED SCI	Experimental study	[119]
Vulvovaginal atrophy (VVA)	Postmenopausal women	ITALY	146	20.29	2015	Vita-Salute San Raffaele University	MENOPAUSE	Experimental study	[16]
Breast cancer	Patients with breast cancer experiencing treatment-induced menopause	Netherlands	146	14.40	2012	Netherlands Cancer Institute	J CLIN ONCOL	Experimental study	[120]
Breast cancer/Ovarian Suppression	Premenopausal women with stage I to III hormone receptor-positive or hormone receptor-negative breast cancer	ITALY	144	20.29	2015	University of Genoa	JAMA-J AM MED ASSOC	Experimental study	[121]
Osteoporosis	Pre- or early perimenopausal	USA	142	13.60	2012	University of California System	J BONE MINER RES	Observational study	[122]
Cardiometabolic	Menopausal transition	Canada	141	13.80	2012	University of Ottawa	MENOPAUSE	Observational study	[123]
Working memory		USA	137	17.00	2014	Icahn School of Medicine at Mount Sinai	P NATL ACAD SCI USA	In vivo study (animal)	[124]
Chronic inflammatory diseases	Postmenopausal women	ITALY	135	18.00	2015	University of Milan	SCI REP-UK	In vitro study	[125]
Estrogen therapy	Premenopausal women	ITALY	135	14.00	2013	University of Foggia	REDOX BIOL	Observational study	[126]
Cardiovascular disease (CVD)	Age at natural menopause	Australia	135	39.67	2019	University of Queensland	LANCET PUBLIC HEALTH	Observational study	[127]
Vaginal atrophy	Postmenopausal women	Brazil	130	30.25	2018	ABC School of Medicine	MENOPAUSE	Experimental study	[128]
Vulvovaginal atrophy (VVA)	Menopause	Canada	130	21.50	2016	EndoCeutics Inc	MENOPAUSE	Experimental study	[129]
Breast cancer	Postmenopausal women	USA	130	14.00	2013	University of California System	JNCI-J NATL CANCER I	Observational study	[130]
Insulin resistance		USA	129	13.89	2013	Yale University	ENDOCRINOLOGY	In vivo study (animal)	[131]
Venous thromboembolism (VTE)	Postmenopausal women	USA	127	12.50	2012	University of Oxford	J THROMB HAEMOST	Observational study	[132]
Papillary thyroid cancer	Menopause	USA	127	12.20	2012	Georgetown University	J CLIN ENDOCR METAB	Observational study	[133]
Depressive symptoms	Around natural menopause	USA	126	15.50	2014	University of Pennsylvania	JAMA PSYCHIAT	Observational study	[134]
Vulvovaginal atrophy (VVA)	Postmenopausal women	ITALY	122	17.29	2015	University of Palermo	MATURITAS	Experimental study	[135]
Osteoporosis		JAPAN	120	13.00	2013	Keio University	Proc Natl Acad Sci USA	In vitro study(animal)	[136]
Hot flash	Premenopausal at baseline and reached natural menopause	USA	117	14.25	2014	University of Pennsylvania	MENOPAUSE	Observational study	[137]
Breast cancer		UK	117	14.13	2014	University of Sheffield	CLIN CANCER RES	In vivo study (animal)	[138]
Cognitive impairment	Pre-to postmenopause	USA	116	12.11	2013	University of Pennsylvania	J CLIN ENDOCR METAB	Observational study	[139]
Genitourinary syndrome	Postmenopausal women	ITALY	114	16.14	2015	University of Pisa	CLIMACTERIC	Experimental study	[17]
Hot flash and night sweat	Menopause transition and postmenopause	UK	112	11.20	2012	King’s College London	MENOPAUSE	Experimental study	[140]
Vaginal atrophy	Postmenopausal women (aged 55–65 years)	USA	112	12.33	2013	George Washington University	MENOPAUSE	Observational study	[141]
Cardiovascular disease (CVD)		USA	111	13.63	2014	University of New Mexico	SCI REP-UK	In vivo (animal) and in vitro study	[142]
Cognitive impairment	Postmenopausal women (within 6 years of menopause or 10 + years)	USA	110	17.17	2016	Stanford University	NEUROLOGY	Experimental study	[143]
Vasomotor symptoms	Perimenopausal and postmenopausal women	USA	109	13.25	2014	Harvard University	JAMA INTERN MED	Experimental study	[144]
Cognitive impairment	Postmenopausal women	USA	111	11.78	2013	Wake Forest University	JAMA INTERN MED	Experimental study	[145]
Cognitive impairment	Perimenopausal and postmenopausal women	USA	110	20.40	2017	Weill Cornell Medical College	NEUROLOGY	Observational study	[146]
Cardiovascular disease and cancer	Postmenopausal women	USA	109	26.25	2018	University of California	MENOPAUSE	Observational study	[147]
Obesity	Menopause transition (MT)(Mean baseline age was 47.1 years (SD, 2.6 years) and average age at FMP was 52.2 years (SD, 2.8 years))	USA	109	31.33	2019	University of California at Los Angeles	JCI INSIGHT	Observational study	[148]
Age at natural menopause (ANM)	Women (40–65 years)	CHINA	107	10.40	2012	Nanjing Medical University	MATURITAS	Observational study	[149]
Cardiovascular Disease、Glucose and lipid metabolism、Osteoporosis	Postmenopausal women	CHINA	106	11.22	2013	Wuyishan Municipal Hospital	J CLIN ENDOCR METAB	Observational study	[150]
Glucose and lipid metabolism		JAPAN	106	9.09	2011	Osaka University	AM J PHYSIOL-GASTR L	In vivo study (animal)	[151]
Osteoporosis	Postmenopausal women	USA	104	13.00	2014	University of Virginia	J CLIN ENDOCR METAB	Experimental study	[152]
Depression	Postmenopausal women	USA	104	14.71	2015	National Institutes of Health	JAMA PSYCHIAT	Experimental study	[153]
Osteoporosis	Perimenopausal women	USA	106	10.20	2012	Duquesne University	J PINEAL RES	Experimental study	[154]
Breast cancer	Postmenopausal; premenopausal women; late menopause	Turkey	104	11.22	2013	Ankara Oncology Research and Training Hospital	BREAST	Observational study	[155]
Unilateral oophorectomy、Premature ovarian failure (POF)、Early menopause (EM)	Pre- and postmenopausal women	JAPAN	102	10.00	2012	Tokushima University	MATURITAS	Observational study	[156]
Anxiety	Early or late perimenopause; postmenopause; premenopause	USA	101	11.22	2013	University of Pittsburgh	MENOPAUSE	Observational study	[157]
Bone formation and bone resorption		USA	101	9.60	2012	Mount Sinai School of Medicine	PROC NATL ACAD SCI USA	In vivo study (animal)	[158]
Osteoporosis	Postmenopausal women	ITALY	101	10.56	2013	University of Rome Sapienza	BONE	Observational study	[159]
Premature and early natural menopause	Premature menopause (FMP < 40 years) and early menopause (FMP 40–44 years)	Australia	100	18.60	2017	University of Queensland	HUM REPROD	Observational study	[160]
Primary Ovarian Insufficiency	An Israeli Arab family with POI	Israel	100	12.38	2014	Tel Aviv University	J CLIN ENDOCR METAB	Observational study	[161]
Osteoporosis	Healthy postmenopausal women (aged 55–65 years)	Netherlands	100	11.00	2013	Maastricht University	OSTEOPOROS INT	Experimental study	[162]
Vulvovaginal atrophy (VVA)	Women presenting with GSM (mean age 58.6 ± 8.8 years)	USA	100	16.33	2016	Stanford University	MENOPAUSE	Experimental study	[163]
Vulvovaginal atrophy (VVA)	Postmenopausal women (aged 45–75 years)	ITALY	99	16.33	2016	University of Pavia	CLIMACTERIC	Observational study	[164]
Hot flash		USA	99	9.80	2012	University of Arizona	PROC NATL ACAD SCI USA	In vivo study (animal)	[165]
Genitourinary Syndrome of Menopause (GSM)	Menopause	Argentina	99	19.40	2017	Mendoza University	LASER SURG MED	Experimental study	[18]
Cardiovascular disease (CVD)	Post-menopausal women( The mean age was 64.9 ± 8.9 years)	USA	99	22.50	2018	Johns Hopkins University Bloomberg School of Public Health	J AM COLL CARDIOL	Observational study	[166]
Vulvovaginal atrophy (VVA)	A median age of 58 years (range 45–90 years)	USA	98	18.80	2017	University Hospitals Cleveland Medical Center, MacDonald Women's Hospital	J SEX MED	Observational study	[11]
Age at menopause	Menopausal age was 50 years (range 30.1–58.2 years)	Iran	97	10.78	2013	Research Institute for Endocrine Sciences	J CLIN ENDOCRINOL METAB	Experimental study	[167]
Cognitive function	Premature menopause (aged 40 years)	France; Australia	97	11.88	2014	Université Montpellier 1; Murdoch Children's Research Institute; The University of Melbourne	BJOG-INT J OBSTET GY	Experimental study	[168]
Insomnia	Postmenopausal women(50–65 years old)	Brazil	94	9.10	2012	Universidade Federal de Sao Paulo	MENOPAUSE	Experimental study	[169]
Vulvovaginal atrophy (VVA)	Postmenopausal women; (mean age 53.3 years) + (mean time of menopause 6.6 years)	ITALY	94	15.17	2016	Careggi University Hospital	ARCH GYNECOL OBSTET	Experimental study	[170]
Genitourinary Syndrome of Menopause (GSM)	Woman (aged 59.3 ± 7.4 years)	ITALY	93	15.17	2016	University of Modena and Reggio Emilia	MATURITAS	Observational study	[12]
Osteoporosis	Early menopause(aged 48 years at the start of the study)	SWEDEN	93	9.10	2012	Lund University	BJOG-INT J OBSTET GY	Observational study	[171]
Menopausal transition	Menopausal transition(Women aged 42–52 years)	USA	91	8.70	2012	University of Pittsburgh	J CLIN ENDOCRINOL METAB	Observational study	[172]
Cardiovascular disease (CVD)/Atherogenic profile	Premenopausal women and men, postmenopausal women and men	UK	90	12.57	2015	Imperial College London	MATURITAS	Observational study	[173]
Nonalcoholic fatty liver disease (NAFLD)	Premenopausal women	JAPAN	89	8.70	2012	Immunology Frontier Research Center at Osaka University	WORLD J GASTROENTEROL	Observational study	[174]
Vascular endothelial function	Postmenopausal women	JAPAN	89	8.60	2012	University of Tsukuba	NUTR RES	Experimental study	[175]
Hepatic fibrosis	Postmenopausal women	USA	89	10.00	2016	University of Arkansas	HEPATOLOGY	Observational study	[176]
Breast cancer	Menopausal women	France	87	9.67	2013	University Paris-Sud	PLOS ONE	Observational study	[177]
Age at natural menopause (ANM)	Age at Natural Menopause	USA	87	9.56	2013	University of California at San Francisco	CANCER	Observational study	[178]
Cognitive Performance, Mood and Cerebrovascular Function	Postmenopausal women	Australia	87	9.60	2017	University of Newcastle	Nutrients	Experimental study	[179]
Trabecular bone score (TBS)	Women(aged 45–85 years)	France	87	6.71	2015	University of Southern California	MENOPAUSE	Experimental study	[180]
Bone mineral density (BMD)	Breast cancer survivors(mean age, 46.5 years)	USA	87	5.00	2013	Oregon Health & Science University	Osteoporos Int	Experimental study	[181]
Sleep disorder	Women (aged 40–59 years)	Latin America	86	3.60	2012	Collaborative Group for Research of the Climacteric in Latin America	Maturitas	Observational study	[182]
Ovarian reserve	Women (aged 21–41 years)	Denmark	86	2.78	2013	Copenhagen University Hospital	J Clin Endocrinol Metab	Observational study	[183]
Ovarian reserve	Women (aged 18–45 years) with brca mutations	USA	85	5.11	2013	Rhône-Durance Clinic	Osteoporos Int	Observational study	[184]
Subclinical atherosclerosis across menopause strata	Postmenopausal women	USA	85	7.38	2014	Cedars-Sinai Medical Center	Fertil Steril	Experimental study	[185]
Gut associated inflammatory and autoimmune disorder	Women (aged 20–60 years)	USA	85	6.11	2013	University of CA Davis Health System	Biol Sex Differ	In vitro study	[186]
Breast cancer	Postmenopausal women	USA	84	9.00	2016	Channing Division of Network Medicine	AM J EPIDEMIOL	Observational study	[187]
Intima-media thickness (IMT) and adventitial diameter (AD)	Women (aged 42–57 years)	USA	84	5.44	2013	University of Pittsburgh	MENOPAUSE	Observational study	[188]
Menopausal transition's health symptoms	Women (aged 47-54 years) who experienced natural menopause	UK	84	3.33	2013	University College and Royal Free Medical School	BMJ	Observational study	[189]

### Active authors

**Fig. 1:**
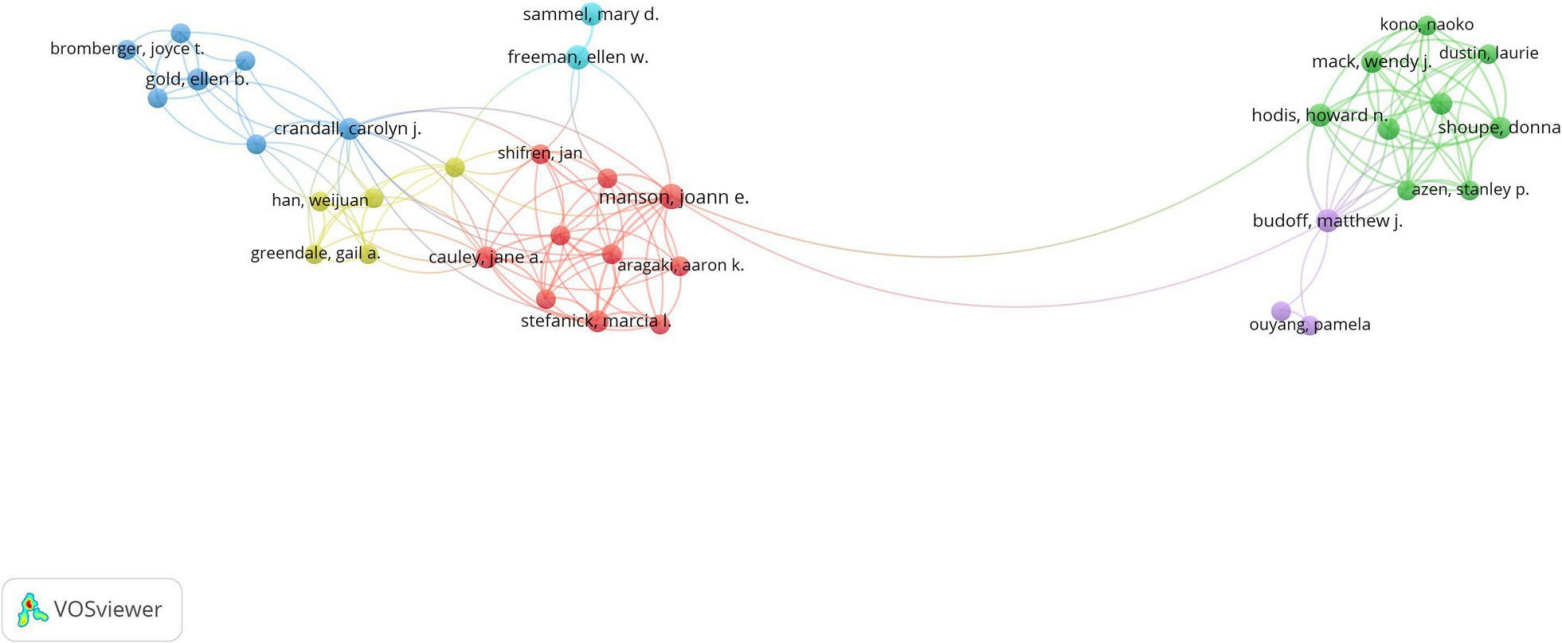
The network of authors engaged in 100 top-cited MPS research

In order to finding the information on influential author and research groups, the fruitful partnership network of authors was constructed (Fig. 1). Of the top 2 active authors (published above 3 articles), Manson, joann e. contributed the most papers (6 publications, 5%), focusing on MPS related biological aging, breast cancer, cardiovascular risk factors, vasomotor symptoms, cognitive function, vaginal estrogens. Followed by Freeman, ellen w., Hodis, howard n., Budoff, matthew j. with 5, 4 and 4 publications respectively, which also paid more attention on the anxiety or depression on the above MPS related topic. We conducted correlation analyses to assess the relationships between journal impact factors and both citation frequency and average citations per article. Initial normality tests for these variables revealed that they did not follow a normal distribution (*P* < 0.000), thus Spearman's rank correlation was utilized. A weak positive correlation was identified between journal impact factors and average citation counts (*r* = 0.258, *P* = 0.010), and a low-to-moderate positive correlation between journal impact factors and citation frequency (*r* = 0.310,* P* = 0.002).

### Active countries and institutions

**Fig. 2:**
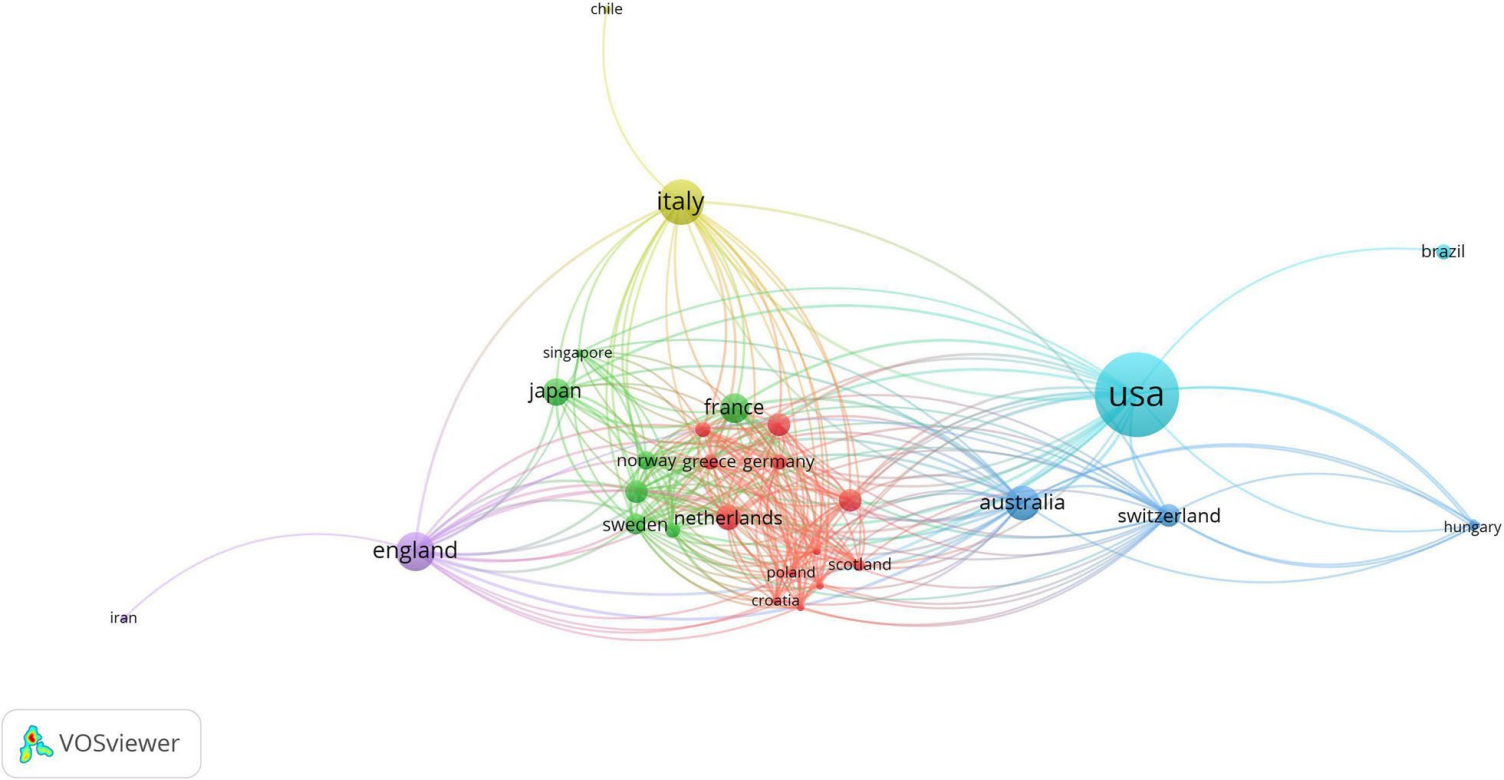
The network of countries engaged in 100 top-cited MPS research

**Fig. 3:**
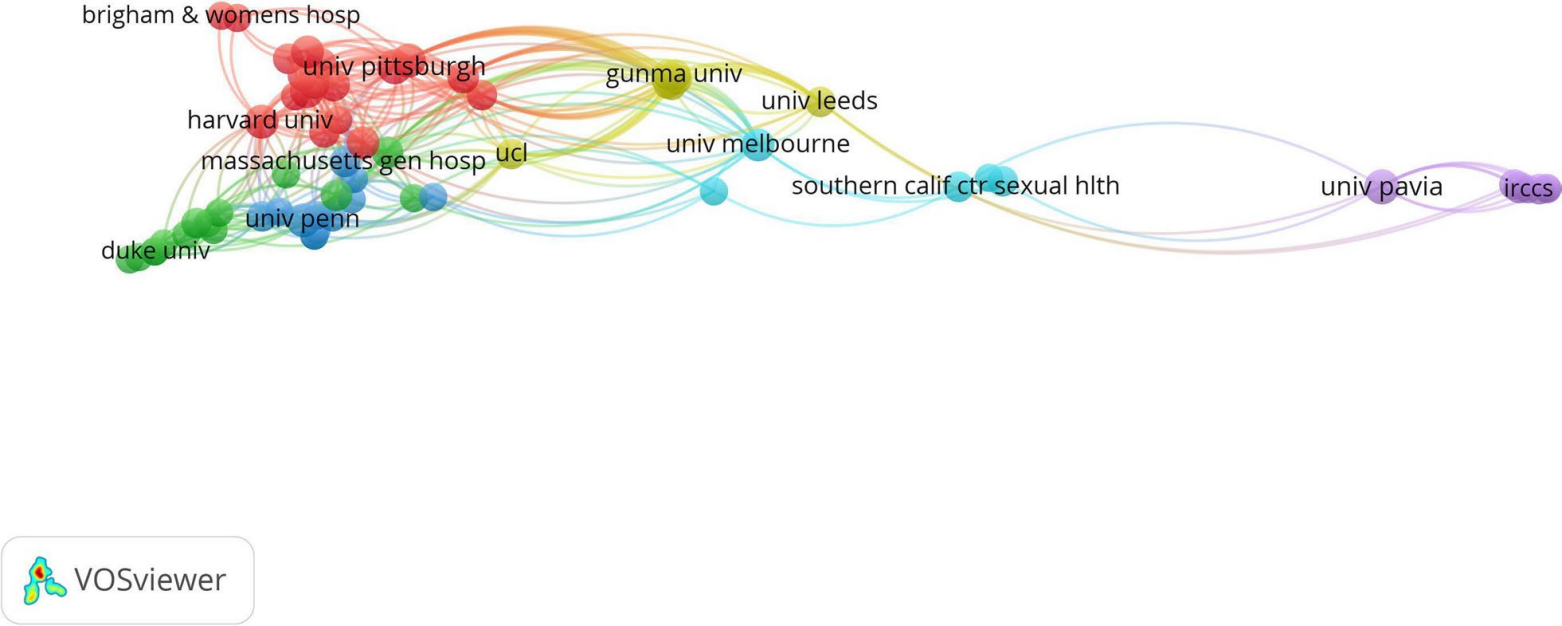
The network of institutions engaged in 100 top-cited MPS research

The partnership network of countries and institutions was constructed. In the database, with established good cooperative relations with scientists from many countries, the leading country was the USA, which published 48 articles, followed by Italy (n = 19), England (n = 15), and Australia (n = 12) (Fig. 2). As the most productive institution,University of Pittsburgh has been published 9 articles (Fig. 3). Followed by Università degli Studi di Pavia (n = 8), Harvard University (n = 7) and Stanford University(n = 7).

### Active Keywords

**Fig. 4:**
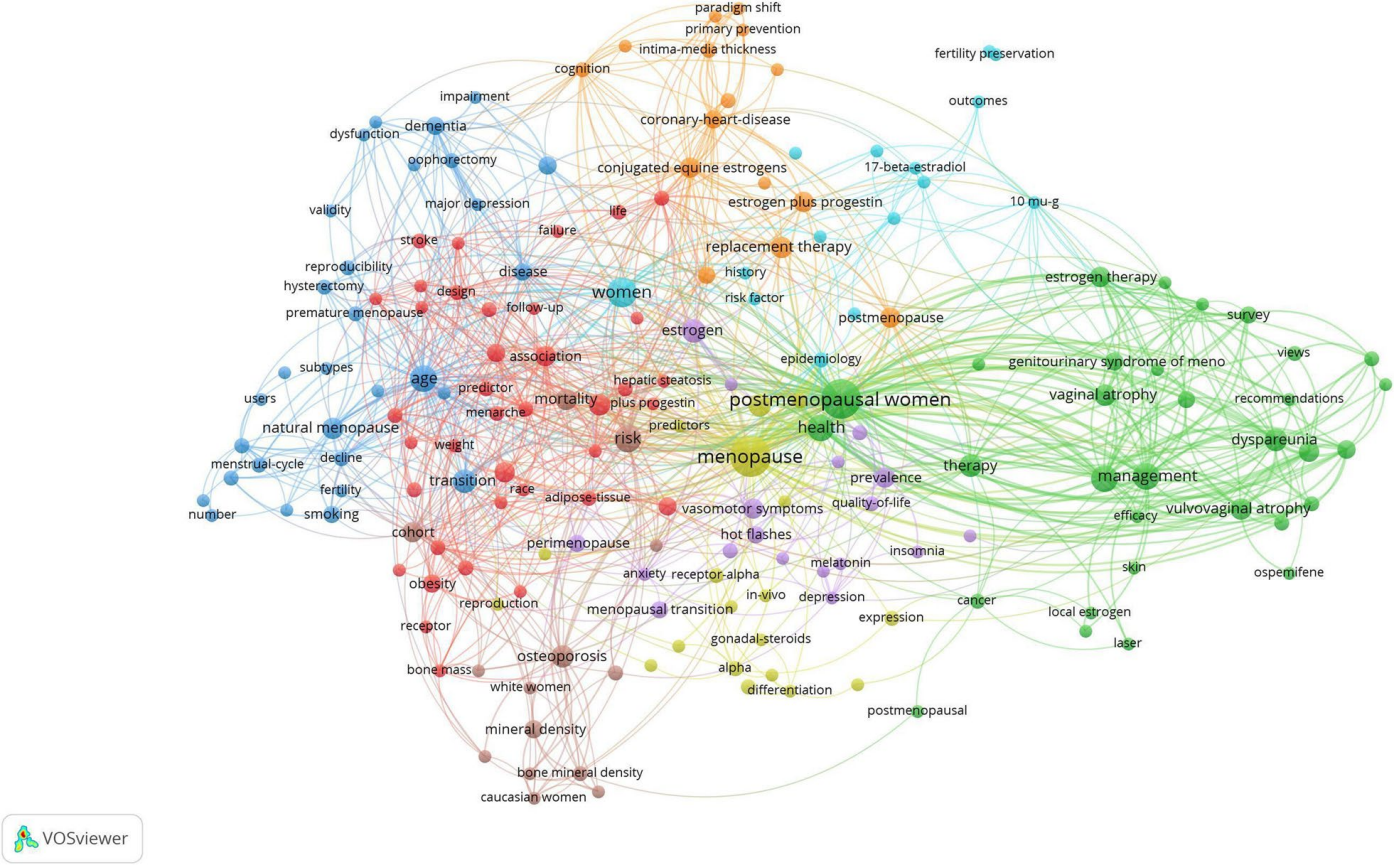
The network of keywords engaged in 100 top-cited MPS research

**Fig. 5:**
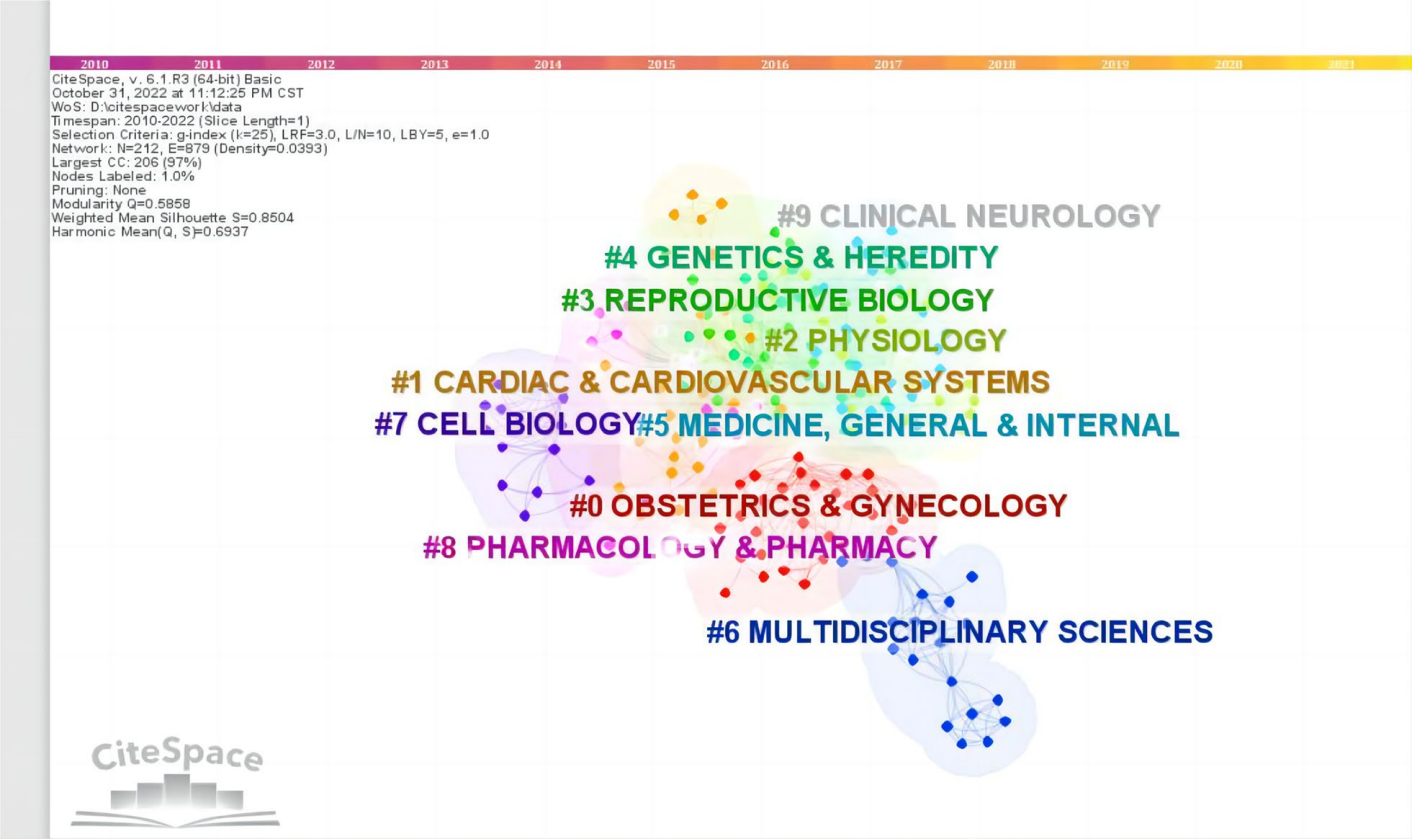
The network of classification engaged in 100 top-cited MPS research

**Fig. 6:**
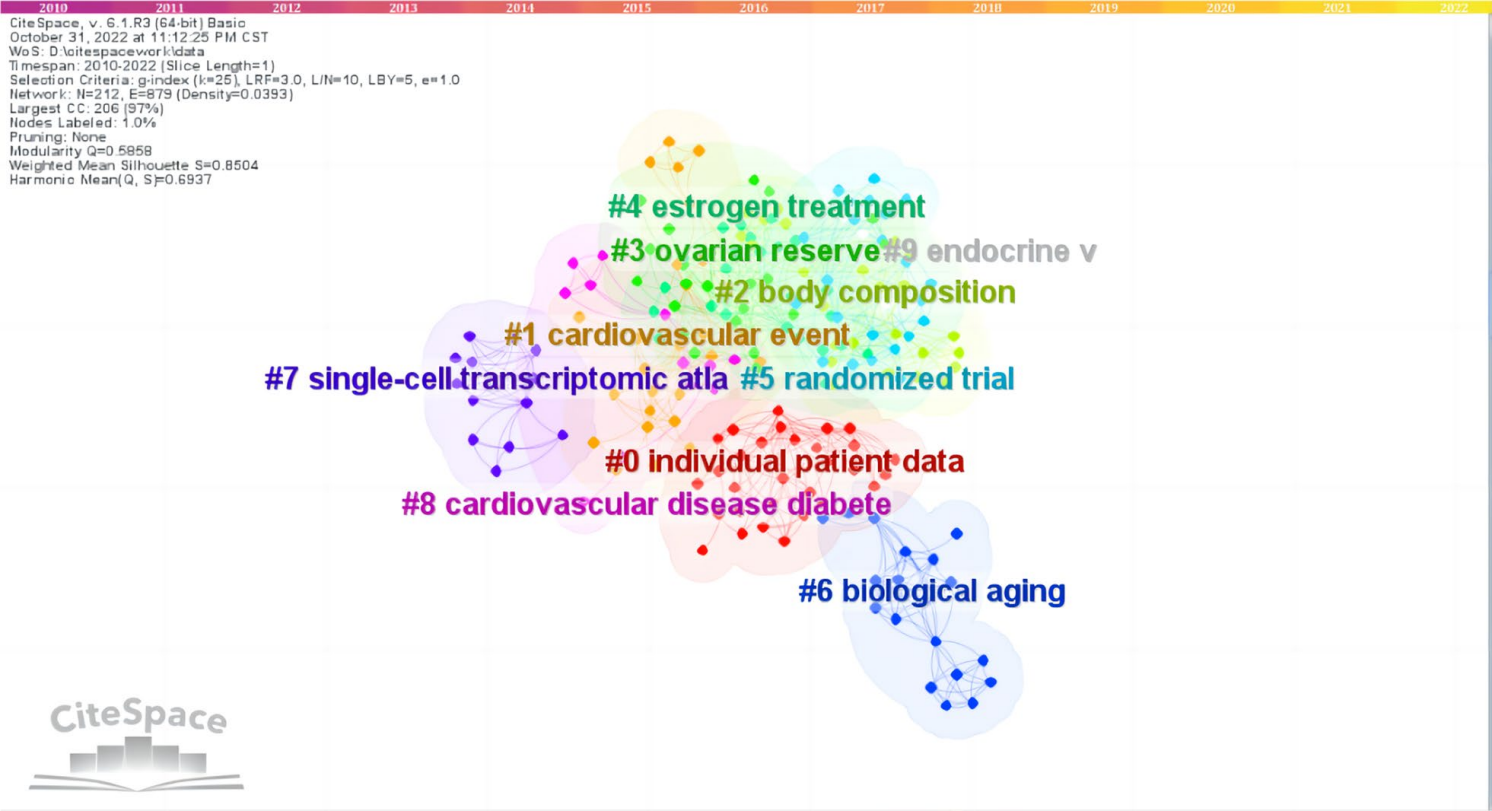
The network of title engaged in 100 top-cited MPS research

CiteSpace 5.8.R3 was used to construct the keywords analysis, which showed a reasonable description of research focus in MPS. Of the 10 types in keywords, the top 5 types were interlace, genitourinary syndrome of menopause, bone mineral density (BMD), anti-mullerian hormone (AMH), loci (Fig. 4). Of the 10 types in classification, the top 5 types were obstetrics and gynecology, cardiac and cardiovascular systems, physiology, reproductive biology, genetics and heredity (Fig. 5). Of the 10 types in title, the top 5 types were individual patient data, cardiovascular event, body composition, ovarian reserve, estrogen treatment (Fig. 6). These involve the prominent problems from the vasomotor symptom (VMS) in MPS to vulvovaginal atrophy and genitourinary syndrome of menopause in menopausal related diseases(Table 2).

**Table 2:**
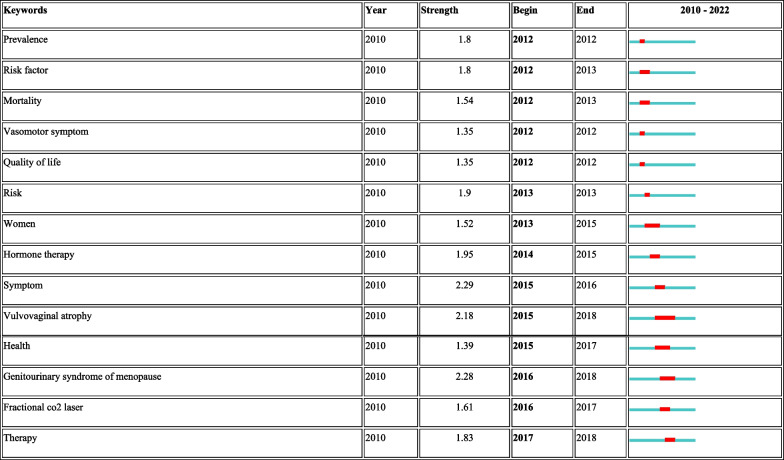
Top 14 keywords with the strongest citation bursts

## Discussion

MPS has developed into a global health epidemic. Under the circumstances that the aging society has formed and gradually intensified, it will suffer more chronic diseases especially in countries and regions with high life expectancy. In particular, the post-menopause time are expected on average to live more than another 30 years. In this bibliometric study and systematic review of MPS related top-cited articles, the analysis showed the information of authorship, country, institution and keywords. It systematically summarized the development status in this field in the past 10 years in order to provide ideas for future research.

### Genitourinary syndrome of menopause

The condition of hypoestrogenism related to menopause has a strong negative impact on vaginal and urinary health, often leading to a condition called genitourinary syndrome (GSM), a term put forward in 2014 [7], although it was used to be called vulvar and vaginal atrophy (VVA). Approximately 50% of postmenopausal women have vulvar, vaginal, and clitoral symptoms, including vaginal dryness, irritation, and itching; sexually related problems such as dyspareunia; and urinary problems including dysuria and recurrent urinary tract infections [8, 9]. The most recent surveys indicated that VVA affected most peri- and postmenopausal women with a prevalence ranging from 36% to almost 90% [10], so GSM is a common, under-diagnosed, and under-treated disorder [11, 12]. Measures to improve its early detection and its appropriate management are needed. However, significant barriers to treatment include a lack of knowledge about VVA, reluctance to discuss symptoms with healthcare professionals (HCPs), safety concerns, inconvenience, and inadequate symptom relief from available treatments [13].

People pay more attention to non-hormonal therapy and newer treatment options including selective estrogen receptor modulators, vaginal dehydroepiandrosterone, and laser therapy, etc. Treatment with the fractional CO2 laser has been proven to be feasible and induced a significant improvement of VVA symptoms by ameliorating vaginal health in postmenopausal women as well as a significant improvement of sexual function and satisfaction with sexual life [14, 15]. Its security has also been assessed, which indicated that it could produce a remodeling of vaginal connective tissue without causing damage to surrounding tissue [16]. Vaginal erbium laser is the second-generation thermotherapy for the genitourinary syndrome of menopause, and recent data suggested that laser energy can be used for the treatment of postmenopausal women suffering from GSM with rapid and more long-lasting effects compared to topical estriol treatment [17, 18].

### Menopausal hormone therapy

The most-cited article was published by Schierbeck, Louise Lind from Denmark published in BRITISH MEDICAL JOURNAL in 2012 (cited 397 times) [19]. It focus on hormone replacement therapy (MHT). Although the situation has changed a lot since the 1942 approval of conjugated equine estrogens, menopausal hormone therapy (MHT) remains the most standard scheme to collectively address the menopausal related symptoms (such as vasomotor symptoms) and additional concerns (such as postmenopausal osteoporosis) [20]. However, including oestrogen or an oestrogenic compound, MHT is also associated with risks of serious health conditions including breast, ovarian and endometrial cancer, stroke and venous thromboembolism, etc.. Additionally, MHT related health risks are proportional to the duration of use. It should be used in before the average menopause age, not all age groups [21, 22]; and racial differences should be taken into account [23, 24]. Although the development of transdermal or vaginal drug delivery has greatly reduced the side effects, the acceptance of menopausal suffers is still low [25, 26]. Therefore, the optimal duration of MHT cannot be recommended, but the initial indications of MHT and the balance of interests and risks for each woman must be strictly considered (Mendoza et al. 2022; Trémollieres et al. 2022; Velentzis et al. 2021).

In recent years, studies have conducted in-depth research on the mechanism of adverse events (AEs) caused by MHT. Dydrogesterone has displayed a favorable safety and tolerability profile during its 60-year use. AEs concerning breast cancer risk, endometrial cancer risk, venous thromboembolism risk, and cardiovascular risk were found to be minimal when dydrogesterone was used as part of a menopausal hormone therapy regimen lasting ≤ 260 weeks [27]. Over a (median) 18-year follow-up period (1993–2016), conjugated equine estrogens nominally significant reduce for coronary heart disease, breast cancer, hip fracture, and all-cause mortality. However, estrogens plus progestin increased breast cancer risk, and reduced endometrial cancer risk [28]. With fewer AEs and more benefits in better sexual life, skin condition and physical activity, alternative therapies receives more and more attention [29]. Compared with, medroxyprogesterone acetate (MPA) and norethisterone (NET) which increased breast cancer risk, evidence suggests a differential effect of MHT containing E2 or natural progesterone (P4) and those containing CEE or progestins, with some evidence trending to a potentially better safety profile with E2 and/or P4 [30–32]

### Cardiovascular disease

The decrease in estrogen level causing the dysfunction of the autonomic nervous system and the decrease of the protective effect on blood vessels, which increases the vasodilation and contraction symptoms that may adversely affect vascular health as well as the occurrence of cardiovascular diseases [33], such as atherosclerosis, stroke, hypertension, and arrhythmias.In the cardiovascular system, aging is accompanied by increased stiffness, increased fibrosis, loss of contractile reserve, increased ROS and endothelial dysfunction. All of these factors contribute to cardiovascular dysfunction [34]. Endothelial dysfunction, on the other hand, is characterized by reduced endothelium-dependent vasodilation, a biomarker of aging, and an important predictor of cardiovascular events in women [35]. Menopausal hormone therapies (MHT) may modulate endothelial function and reduce development of vascular lesions.

Sex hormone levels, especially those in which total testosterone is higher compared to estrogen in the postmenopausal [36], that is, lower endogenous estrogen levels resulting from menopausal transition [37] and higher endogenous androgens [38], may mediate the increased risk of cardiovascular disease (including blood pressure, C-reactive protein (CRP), and insulin resistance) in postmenopausal women [39].The Danish Osteoporosis Prevention Study (DOPS) randomized trial found that women treated with estradiol or in combination with sequential norethindrone acetate in the early postmenopausal period had a significantly lower risk of coronary heart disease than untreated women, demonstrating the effectiveness of hormone replacement therapy for cardiac diseases such as heart failure or myocardial infarction in perimenopausal women [40].The Early versus Late Intervention Trial with Estradiol (ELITE) suggests that the effect of hormone therapy on cardiovascular disease varies may depend on the timing of initiation of treatment relative to the perimenopausal period. Carotid intima-media thickness (CIMT) has been found to be inversely correlated with plasma total estradiol levels in the late postmenopausal period [41]. Early Menopausal hormone therapy (MHT) did not affect progression of atherosclerosis despite improving some markers of CVD risk [42]. Oral estradiol therapy within 6 years of postmenopausal significantly reduced CIMT progression but not when it was initiated in women who were 10 or more after menopause [41].Therefore, grasping the duration of menopause is helpful for the prevention and treatment of cardiovascular disease risk in menopausal women.

### Breast cancer

In 2020, female breast cancer ranked first in the global incidence of malignant tumors, with about 2.3 million new cases, accounting for 11.7% of all new cases [43]. Numerous studies have shown that women have an increased risk of breast cancer after menopause. Changes in hormone levels are the underlying cause of the risk of disease and decreased serum luteinizing hormone and follicle-stimulating hormone levels after menopause are associated with increased ER and PR expression and decreased HER2 expression in breast cancer patients [44, 45]. Total testosterone TT, testosterone BT, dehydroepiandrosterone, and estradiol increase the risk of different subtypes of BCs [46]. Obesity is one of the main risk factors causes especially an increased incidence of ER + , low-grade breast cancer [47, 48]. Obesity-induced inflammation plays a role in the development and progression of breast cancer, and plasma levels of measured inflammatory biomarkers are positively associated with breast cancer risk in postmenopausal women, but the association between measured inflammatory biomarkers and breast cancer risk did not vary by breast cancer subtype [49], and serum uric acid plays an important mediating role in the obesity-breast cancer relationship [50, 51]. MHT use was associated with a lower risk of breast cancer mortality following surgical menopause before 45 years, at 45–49 years or at ≥ 50 years, and the association between MHT use and the risk of breast cancer mortality did not differ by MHT use duration (< 6 or 6–20 years). MHT use was also associated with a lower risk of breast cancer mortality following natural menopause before 45 years or hysterectomy before 45 years [52]. MHT use was associated with an increased risk of breast cancer (odds ratio [OR] 1.12, 95% CI 1.09–1.15). And, this risk appears predominantly mediated through formulations containing synthetic progestins. micronized progesterone may be the safer progestogen to be used [53–55]. Nearly half of the effect of MHT on the risk of hormone receptor-positive BC is mediated by breast density. Indicate that we should use MHT with caution for women [56, 57]. cCNE2 may be a candidate gene for estrogen-progestin-induced breast cancer. But strong evidence is still lacking to support for common genetic variants altering the effect of MHT on breast cancer risk in estrogen-progestin MHT combinations or in estrogen receptor (ER) positive cases [58, 59]. The gut microbiota can influence breast carcinogenesis, but it remains to be investigated whether there are differences in the abundance, diversity, and composition of the gut microbiota between postmenopausal breast cancer patients and controls [60, 61].Lack of physical activity, high postmenopausal body mass index, alcohol consumption, and use of menopausal hormone therapy are known risk factors for breast cancer [62–64]. There was a definite association between modifiable lifestyle factors and 10-year all-cause mortality. The strongest association was found between BMI ≥ 30 kg/m2 and all-cause mortality compared with BMI 18.5 ~ 25 kg/m2 [HR (95% confidence interval, 1.19,1.06 ~ 1.34)]. There was no evidence of associations between modifiable risk factors and 10-year mortality differed by subtype [65, 66].

### Osteoporosis

Osteoporosis is recognized as the most common complication of menopause, which closely related to fracture and osteodynia [67]. A new discovery showed that hearing and body balance at baseline exceeded initial BMD in predicting incident fractures in menopausal women with osteoporosis regardless of treatment during 25-year follow-up [68, 69]. Genome-wide association studies (GWAS) identified 12 loci that were significantly associated with BMD at any site in Chinese population, and rs1239055408 G > GA (KCNJ2) was associated with BMD only in women [70]. And, High-fat diet-induced obesity augments the deleterious effects of estrogen deficiency on bone, which resulting in accelerated cellular senescence, expansion of BMAT and impaired bone formation leading to decreased bone mass [71]. Longer leukocyte telomere length (LTL) was weakly associated with reduced risk of any incident fracture in women, however with less evidence in men [72]. Additionally, with a higher bone loss rate in surgically induced menopausal women compared with a natural process, MHT could significantly suppress the high bone remodeling [73]. In a Women's Health Initiative (WHI) hormone therapy trials including 25,389 postmenopausal women aged 50–79 years, MHT reduced the risk of fracture regardless of baseline FRAX fracture probability and falls history [74]. The MHT scheme of percutaneous estradiol gel (1.5 mg/day) plus oral micronized progesterone (100 mg/day) for 4 years has a low probability in fracture recurrence and mortality [75]. Erxian Decoction (EXD), as a commonly used alternative therapy of traditional Chinese medicine, is exerted therapeutic effects for OP through multiple functional signal pathways [76]. Phytoestrogens represented by the nanoparticles of betulinic acid (BA/NPs), as an naturally occurring PPAR-γ inhibitor, has great potential to improve osteoporosis in the in vivo and in vitro model [77–79].

### Cognitive impairment

E2 is important for regulating hippocampal learning and memory. With the increase of subjective cognitive dysfunction and elevated rates of depression in this period, premature estrogen decline can lead to mild cognitive impairment (MCI), even Alzheimer's disease (AD), dementia with Lewy bodies (DLB) and vascular dementia (VaD) [80, 81]. However, as continued interest and debate, whether the MHT cure or prevent cognitive impairment or not. Recent research shows that MHT to improve cognitive functioning has only a few scenarios where it would be recommended and that particular caution may be warranted for carriers of the APOE ε4 allele [82]. Additionally, women at genetic risk for AD (APOE e4 allele carriers) have particularly shown favorable results from MHT treatment [83]. A meta-analysis including 10 RCTs with 2,818 participants showed that current available evidence does not support MHT had no effect on verbal memory in postmenopausal women, and also may impair some domains of short-term memory [84]. Further research findings that it has shown a slightly increased risk of developing AD among long term users of oestrogen-progestogen therapies, rather than taking oestrogen-only therapy [85]. A new perspective study of Functional cerebral asymmetry (FCA) discovered that right hemisphere is mainly affected by aging, and hormonal modulation improves the interplay between the two hemispheres and reduces FCA [86]. And also, a PET Study showed post-menopausal women showed significantly higher tau-PET signal in parieto-occipital regions, but were not moderated by Aβ burden or APOEε4 [87]. Additionally, following lower side effects compared with MHT, phytoestrogens as neuroprotective agents or epigenetic modifiers recover and maintain cognitive functions [88, 89]. But, very little is known regarding the regulation of synaptic plasticity genes, and also the precise regulation mechanism needs to be further explored. Moreover, postmenopausal status and a family history of dementia were more frequent among women who had had COVID-19 [90].

### Glucolipid metabolism disorder

Changes in hormone levels lead to abnormalities in lipid metabolism with elevated serum total cholesterol, LDL cholesterol, apolipoproteins, and triglycerides, and decreased high-density lipoprotein cholesterol (HDL-C) [91]. Reduced energy expenditure due to reduced lipid oxidation and reduced leptin sensitivity in menopausal women [92]. Postmenopausal women have a lower gut microbiome diversity, slightly similar to that of men, and are involved in sex hormone retention. One study found that the gut microbe Bacteroides fragilis contributes to obesity in perimenopausal women by suppressing acetic acid levels [93, 94]. In addition, overweight or obese women have more severe and moderate menopausal symptoms [95]. Central adipose tissue accumulation, production of cytokines, and other factors contribute to an increased risk of developing T2DM by causing low-grade systemic inflammation and insulin resistance [96]. The earlier the age of menopause, the higher the risk of developing type 2 diabetes mellitus in women. Autoimmune destruction of follicles, insulin deficiency, and exogenous hyperinsulinemia in diabetic patients disrupt the normal function of the female reproductive system. Making women with diabetes experience menopause earlier [97, 98]. Early hormone therapy in menopausal women improves BMI and lipid levels in women.

## Conclusion

Using bibliometrics, we conducted an analysis to identify the inadequacies, traditional focal points, and potential prospects in the study of MPS across current scientific areas. Treatment and complications are at the core of MPS research, whereas prediction and biomarkers have less literature of high quality. There is a necessity for innovative analytical metrics to measure the real effect of these papers with a high level of citation on clinical application.

## Data Availability

The datasets used or analysed during the current study are available from the corresponding author on reasonable request.
