# Correction: The 100 top-cited articles in menopausal syndrome: a bibliometric analysis

**DOI:** 10.1186/s12978-024-01807-z

**Published:** 2024-06-21

**Authors:** Zishan Jin, Chuanxi Tian, Mengjiao Kang, Shiwan Hu, Linhua Zhao, Wei Zhang

**Affiliations:** 1 Beijing University of Chinese Medicine, Beijing, 100029 China; 2 Institute of Metabolic Diseases, Guang’anmen Hospital, China Academy of Chinese Medical Sciences, Beijing, 100053 China; 3 Gansu University of Chinese Medicine, Lanzhou, 730000 Gansu China

**Correction** **:** **Reprod Health 21, 47 (2024)**

**https://doi.org/10.1186/s12978-024-01770-9**

After publication of this article [1], the authors reported that in the Funding section, an incorrect grant number was given; it has been removed.

The original article has been updated by the authors.
